# Going visible: high-resolution coherent Raman imaging of cells and tissues

**DOI:** 10.1038/s41377-018-0119-5

**Published:** 2019-01-16

**Authors:** Richard C. Prince, Eric O. Potma

**Affiliations:** 1 Department of Biomedical Engineering, University of California, Irvine, CA 92697 USA; 2 Department of Chemistry, University of California, Irvine, CA 92697 USA

**Keywords:** Multiphoton microscopy, Super-resolution microscopy

## Abstract

A simple change of light source might prove what is needed for high-resolution label-free mapping of thin biological samples.

Coherent Raman scattering (CRS) is a relatively young technique in the toolbox of the microscopist. It is a useful imaging approach that enables the generation of images through endogenous chemical species already present in samples, such as biological tissues. However, coherent Raman scattering has not kept up with recent advances in resolution in fields like fluorescent microscopy where the diffraction limit is often exceeded. In their recent work, Bi and colleagues present an extension of CRS microscopy that introduces unprecedented level of detail for CRS imaging of biological specimen^1^.

The development of CRS microscope tracks very closely with the evolution of the laser itself. The first coherent Raman scattering microscope was powered by visible dye lasers, which were common in the early 1980s^2^. The wide spectral tuning range of dye lasers made them a natural choice for CRS experiments. The modern version of the CRS microscope makes use of more reliable and compact ultrafast light sources that provide pump and Stokes beams in the near-infrared (NIR) range^3^. However, the choice of excitation wavelength is predicated on more than just the available laser sources of the day. NIR excitation beams experience less scattering than their visible counterparts, allowing for deeper imaging in turbid media, such as biological tissues. Because the absorption of light by biological materials is significantly higher in the visible range, the use of NIR excitation limits the deposition of energy in samples, thereby reducing photodamaging effects.

Because of their favorable properties for biological imaging, NIR light sources have become the gold standard for CRS. Utilizing NIR excitation, CRS microscopy has been immensely successful at visualizing live cells and tissues, helping unravel outstanding problems in biology and biomedicine. However, the longer wavelength of the NIR sources has a downside. The spatial resolution in optical microscopy is inversely correlated with the wavelength of light; a longer wavelength implies a lower resolution. Using high numerical aperture (NA) objective lenses, the lateral spatial resolution in CRS imaging is typically ~0.3 μm, i.e., too low to resolve many relevant cellular structures.

Encouraged by successes in fluorescence microscopy, many research teams have sought to increase the resolution of CRS microscopy. But unlike fluorescence imaging processes, the efficient population and depopulation of relevant molecular eigenstates, which is required for most super-resolution techniques, is more difficult to accomplish through coherent Raman scattering. Thus far, this line of research has been limited to mostly theoretical exercises^4,5^. Afew recent successes notwithstanding^6,7^, CRS imaging of biological samples at super-resolution remains an elusive goal.

Instead of employing advanced excitation schemes, a straightforward route toward achieving higher resolution is to shorten the excitation wavelength while maximizing the NA of the focusing lens. This strategy has previously been implemented in a CRS microscope for the visualization of gold nanospheres, reaching a lateral resolution of 140 nm^8^. Bi et al. extended this strategy for biological imaging. By using visible pump and Stokes beams and a 1.49 NA objective lens, they achieved stimulated Raman scattering (SRS) imaging of single cells and tissues at a resolution of 130 nm^1^. In doing so, Bi et al. were able to resolve cellular structures previously unseen in CRS microscopy, opening up a wide range of potential imaging studies.

Along with the higher resolution, the shorter wavelength of the excitation beams also brings the driving frequencies closer to the electronic transitions of many endogenous compounds. For vibrational modes that are coupled strongly to such electronic transitions, the corresponding coherent Raman response is significantly enhanced (Fig. 1). This effect, well known in the coherent anti-Stokes Raman scattering (CARS) and SRS spectroscopy literature^9,10^, provides CRS with a considerable boost, allowing the generation of strong signals at lower power.

The work by Bi et al. shows that simple approaches can produce impressive results. Although somewhat limited by tissue scattering and photodamage, CRS microscopy with short wavelength excitation may prove to be a useful tool for mapping biological structures at the 100 nm scales in fixed cells and thin tissue samples. In such studies, resolution is important, and the short wavelength CRS approach constitutes a practical solution for label-free biological imaging.

The resurgence of visible lasers in coherent Raman microscopy brings back echoes of the very first CRS microscope. However, unlike in the 1980s, the required coherent light beams are currently produced by fiber lasers and parametric downconversion techniques. In this regard, the development of high-resolution CRS microscopy is not dependent on dye lasers of yesteryear.

**Fig. 1:**
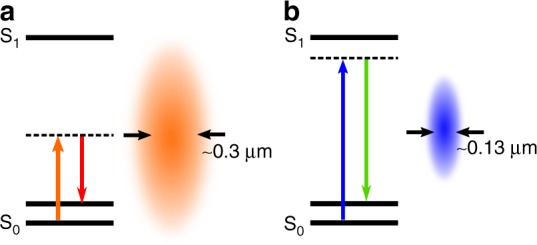
Stimulated Raman scattering (SRS) microscopy with visible laser beams. **a** In most SRS applications, near-infrared excitations beams are used, providing focal excitation volumes with lateral diameters of ~0.3 μm. **b** By using shorter wavelengths, the focal excitation volume is correspondingly smaller, down to 0.13 μm. Additionally, the excitation beams are pre-resonant with a nearby electronic state (S_1_), giving rise to stronger SRS signals
